# Sex Chromosome Differentiation in the Frog Genus *Pseudis* Involves Satellite DNA and Chromosome Rearrangements

**DOI:** 10.3389/fgene.2018.00301

**Published:** 2018-08-07

**Authors:** Kaleb P. Gatto, João V. Mattos, Karin R. Seger, Luciana B. Lourenço

**Affiliations:** Laboratory of Chromosome Studies, Department of Structural and Functional Biology, Institute of Biology, University of Campinas, Campinas, Brazil

**Keywords:** chromosome evolution, heterochromatin, paradoxical frogs, satellite DNA, sex chromosomes

## Abstract

The genus *Pseudis* comprises six frogs of the family Hylidae and only *P. tocantins* had heteromorphic sex chromosomes detected by classical cytogenetics. In this species, the W chromosome is larger than the Z chromosome and has a large heterochromatic block located between the centromere and the nucleolus organizer region (NOR) in the long arm. This large heterochromatic band is enriched for the PcP190 satellite DNA (satDNA), whereas the Z chromosome bears a smaller C-band adjacent to the centromere in the long arm that is not detected by PcP190 probes. To assess sex chromosome differentiation in the genus *Pseudis*, we investigated the PcP190 satDNA in *P. bolbodactyla*, *P. cardosoi*, *P. minuta*, and *P. paradoxa* and in one species of *Lysapsus*, which is the sister genus of *Pseudis*. PcP190 sequences were isolated, sequenced, and the diversity of this class of satDNA was analyzed. To evaluate whether sex-related variations in PcP190 satDNA were present, we used *in situ* hybridization (for *P. bolbodactyla*, *P. paradoxa*, *P. cardosoi*, and *P. minuta*) and Southern blotting analysis (for all species). We found a low level of sex chromosome heteromorphism in *P. bolbodactyla*, as a PcP190 cluster was detected in the short arm of one of the homologs of pair 7 exclusively in females. In *P. paradoxa*, *P. minuta*, and *P. cardosoi*, PcP190 satDNA is not sex-related, although a cluster of PcP190 sequences could be recognized in the NOR-bearing chromosomes 7 of *P. paradoxa* and *P. minuta* and their homologous chromosome 5 of *P. cardosoi*. By tracking cytogenetic data in a species tree, we may hypothesize that the positioning of the PcP190 site adjacently to the NOR (as observed in the long arm of the W chromosome of *P. tocantins*) is a derived condition with respect to the location of the PcP190 site apart from the NOR, in the short arm of the NOR-bearing chromosomes 7 (as present in *P. bolbodactyla*, *P. paradoxa*, and *P. minuta*) or 5 (as present in *P. cardosoi*) and we discuss about the emergence of PcP190 satDNA as a sex-related trait.

## Introduction

In animal and plant species with genetic sex determination, sex chromosomes bear the gene responsible for sex determination (review in Graves, 2008; Ellegren, 2011). Sex chromosomes have an autosomal origin, and after the acquisition of a sex determination locus, recombination between the homologs is suppressed in the sex-specific region, leading to chromosome differentiation, which involves a progressive degeneration of chromosome Y/W (Muller, 1964; Ohno, 1967; review in Ellegren, 2011). Usually, during the differentiation of sex chromosomes, rearrangements (e.g., inversions) and accumulation/loss of heterochromatin segments occur (reviewed by Graves, 2008; Bergero and Charlesworth, 2009). The heterochromatin regions in eukaryotes are mainly populated by satellite DNA (satDNA), which is a class of repetitive sequences characterized by a tandem arrangement with highly repetitive monomeric units longer than 100 bp (reviewed by Charlesworth et al., 1994; Plohl et al., 2012). The accumulation of satDNA in sex chromosomes has been reported for several species of animals and plants (e.g., Nakayama et al., 1994; Hobza et al., 2006; Mariotti et al., 2009), and in addition to being a possible cause of recombinatory suppression, it may also be a consequence of this process (Singh et al., 1976, 1980; Charlesworth et al., 2005; Schartl et al., 2016).

Among anurans, heteromorphic sex chromosomes are rare, but a considerable number of species that bear this feature exhibit a Y or W chromosome with heterochromatin accumulation (Schmid et al., 2012; Schartl et al., 2016). One of those species is *Pseudis tocantins*, which possesses a ZZ/ZW sex determination system, with the W chromosome being submetacentric and larger than the metacentric Z, mainly because of heterochromatin amplification in the long arm of the W chromosome (Wq) (Busin et al., 2008). *Pseudis tocantins* Wq heterochromatin is enriched for the PcP190 satDNA (Gatto et al., 2016), which is a satDNA inferred to have originated from 5S rDNA (Vittorazzi et al., 2011) and has been shown to be present in several species of the superfamily Hyloidea (Vittorazzi et al., 2014; Gatto et al., 2016). An interesting feature of the repetitive units of this satDNA is the presence of a more conserved region (CR), which is very similar among all the taxa, and a hypervariable region (HR) that allows for the identification of several sequence groups (Gatto et al., 2016). In the *P. tocantins* karyotype, the only cluster of PcP190 detected by *in situ* hybridization of PcP190 probes is in the Wq, and Southern blotting analysis using PcP190 probes also allowed for the discrimination between males and females (Gatto et al., 2016).

Besides *Pseudis tocantins*, the hylid genus *Pseudis* encompasses five other species [here we considered *P. platensis* as a junior synonym of *P. paradoxa*, based on the adult and tadpole morphological and acoustical studies from Garda et al. (2010) and Santana et al. (2016)] and none of them have sex chromosome heteromorphism detected by classical cytogenetics (Busin et al., 2001, 2008). The sex chromosome pair of *P. tocantins* was hypothesized to be homeologous to chromosome pair 7 in other species of *Pseudis* (pair 5 in *Pseudis cardosoi*) and chromosome pair 7 of *Lysapsus* based on their similar morphology and the presence of nucleolus organizer regions (NORs) (Busin et al., 2006, 2008; Suárez et al., 2013). No information regarding the presence of the PcP190 satDNA in these species is available.

Considering that sex chromosome heteromorphism in *Pseudis tocantins* is related to the PcP190 satDNA, with the aim of investigating sex chromosome evolution in *Pseudis*, in this paper we analyzed the PcP190 satDNA of four other species of *Pseudis* (i.e., *P. bolbodactyla*, *P*. *cardosoi*, *P. minuta*, and *P. paradoxa*) and one species of *Lysapsus*, which is the sister genus of *Pseudis* (for phylogenetic relationships, see Wiens et al., 2010; Duellman et al., 2016). Chromosome mapping of PcP190 satDNA (i) supported the NOR-bearing chromosomes of the *Pseudis* species as homeologous chromosomes, (ii) revealed that sex chromosome heteromorphism is also present in *P. bolbodactyla* and (iii) allowed for the inference of some chromosome rearrangements during the evolution of this genus.

## Materials and Methods

### Species and Chromosome Preparations

We analyzed eight specimens of *Pseudis bolbodactyla*, seven *P. cardosoi*, eight *P. minuta*, ten *P. paradoxa*, and eight *Lysapsus limellum* (see **Supplementary Table 1** for details). The individuals used in this work had their karyotypes described previously by Busin et al. (2001, 2006, 2008), except for two specimens of *P. minuta* (ZUEC 22082 and ZUEC 22096) and one exemplar of *P. bolbodactyla* (ZUEC 22080), which were collected under the authorization issued by the Instituto Chico Mendes de Conservação da Biodiversidade/Sistema de Autorização e Informação em Biodiversidade (ICMBio/SISBIO) (authorization #45183-3) and deposited at the Museu de Zoologia “Prof. Adão José Cardoso”, Universidade Estadual de Campinas (ZUEC-UNICAMP). Individuals previously used by Busin et al. (2001, 2006, 2008) were collected under authorization issued by the Instituto Brasileiro do Meio Ambiente e dos Recursos Renováveis (IBAMA) (process #02001008875/01-11, license #039/03).

**Table 1:** Groups of PcP190 satellite DNA sequences found in *Pseudis* (present work and Gatto et al., 2016) and *Lysapsus* (present work).

Sequence groups	Groups found in *P. tocantins*^1^	Groups found in *P. bolbodactyla*	Groups found in *P. paradoxa*	Groups found in *P. minuta*	Groups found in *L. limellum*	Average similarity in hypervariable region (%)
PcP-1a	X (*N* = 7)	–	–	X (*N* = 3)	X (*N* = 1)	93.74 (*N* = 11)
PcP-1b	X (*N* = 10)	X (*N* = 1)	–	–	–	99.72 (*N* = 11)
PcP-2	X (*N* = 14)	X (*N* = 12)	X (*N* = 6)	–	–	79.83 (*N* = 32)
PcP-3	X (*N* = 3)	–	–	X (*N* = 33)	–	97.19 (*N* = 36)
PcP-5	X (*N* = 4)	X (*N* = 1)	–	–	–	94.08 (*N* = 5)
PcP-7	X (*N* = 14)	X (*N* = 5)	–	–	–	No hypervariable region^2^
PcP-8	–	–	–	–	X (*N* = 14)	91.54 (*N* = 14)
PcP-9	–	–	–	X (*N* = 1)	–	?^3^

**FIGURE 1:**
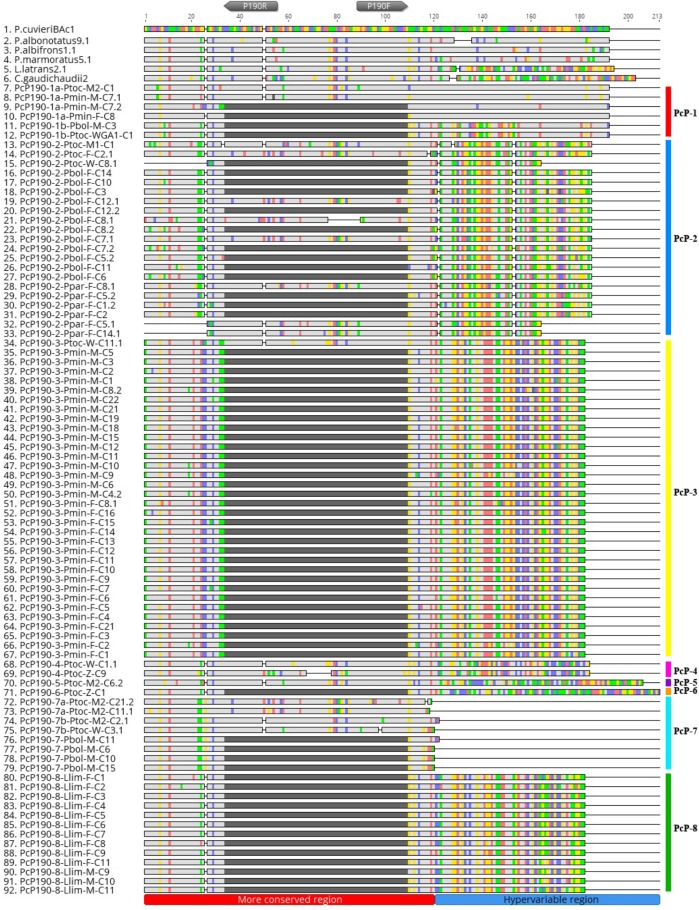
Alignment of PcP190 satDNA sequences isolated from *Pseudis bolbodactyla*, *Pseudis minuta*, *Pseudis paradoxa*, and *Lysapsus limellum* with sequences made available at GenBank for *Pseudis tocantins* (KX170887.1; KX170893.1; KX170895.1; KX170898.1; KX170908.1; KX170911.1; KX170916.1; KX170922.1; KX170924; KX170929.1; KX170930.1; KX170931.1; and KX170933.1), *Physalaemus cuvieri* (JF281121.1; JF281124.1; KM361975.1; KM361678.1; and KM361679.1), *Physalaemus centralis* (KM361684.1 and KM361685.1), *Physalaemus albonotatus* (KM361689.1 and KM361690.1), *Physalaemus albifrons* (KM361694.1; KM361696.1; and KM361698.1), *Physalaemus ephippifer* (KM361699.1 and 361700.1), *Physalaemus marmoratus* (KM361701.1 and KM361703.1), *Leptodactylus latrans* (KM361718.1 and KM361719.1), and *Crossodactylus gaudichaudii* (KM361725.1 and KM361726.1). Gray pentagons indicate the P190F and P190R primer sites. Sequences with a dark gray segment (correspondent to “N” breaks) in the central region of the more conserved region of PcP190 are partial monomers, isolated using the P190F and P190R primers. Sequences with the more conserved region completely sequenced are derived from fragments composed of multiple monomers of PcP190. This figure was created in Geneious 8.0 (Biomatters).

*Monomer lengths and similarity in the hypervariable region are indicated for each group of PcP190 sequences. N, number of sequences. ^1^Based on Gatto et al. (2016). ^2^PcP-7 sequences do not present hypervariable region (**Figure 1**). ^3^Not calculated because only one sequence was available.*

Chromosome preparations were obtained from the cytogenetic collection deposited at the Laboratory of Chromosome Studies of the University of Campinas or directly from the captured specimens of *P. bolbodactyla* and *P. minuta*. In the latter case, the animals were injected intraperitoneally with 2% colchicine solution in distilled water (0.02 mL/g body weight), and after 4 h, they were anesthetized with 2% lidocaine (50 mg/g body weight – cutaneous administration). Chromosome preparations were obtained from the intestine according to King and Rofe (1976), with some minor modifications. Briefly, the intestine was removed from anesthetized animals, cut open lengthwise and incubated for 40 min in a 0.9% sodium citrate solution. The intestine was then placed in a fixative solution of methanol:acetic acid (3:1) and the epithelial cells were vigorously scraped. Cell suspension was centrifuged for 5 min at 800 rpm, the supernatant fixative was removed and the material was washed with new fixative twice. Intestinal cell suspension was transferred to 1.5 mL tube and stocked in a freezer for further use. This protocol was approved by the Committee for Ethics in Animal Use of the University of Campinas (CEUA/UNICAMP) (protocol # 3419-1).

### PcP190 Satellite DNA Isolation and Sequencing

Genomic DNA samples were obtained from liver tissue from male and female individuals of *Pseudis* and *Lysapsus* species (**Supplementary Table 1**) following the TNES method employed by Medeiros et al. (2013). Integrity of genomic DNA was evaluated by electrophoresis in a 0.8% agarose gel and total genomic DNA was quantified in a Nanodrop (Thermo Fisher, United States) spectrophotometer. PcP190 satDNA was isolated by PCR using the primers P190F (5′-AGACTGGCTGGGAATCCCAG-3′) and P190R (5′-AGCTGCTGCGATCTGACAAGG-3′), described previously by Vittorazzi et al. (2011). The PCR program used was: (1) 94°C for 8 min; (2) 39 cycles of 94°C for 30 s, 58°C for 1 min and 72°C for 2.5 min; (3) 72°C for 8 min. PCR amplicons were purified using the Wizard SV Gel and PCR Clean-Up System (Promega) and inserted in a plasmid pGEM-T Easy Vector (Promega). Plasmids were cloned into *E. coli* JM-109 bacteria using the TransformAid Bacterial Transformation Kit (Fermentas), according to manufacturer instructions. Recombinant colonies were selected, plasmid DNA was extracted following Sambrook et al. (1989), and the inserts were amplified by PCR using the T7 and SP6 universal primers. After purification with the Wizard SV Gel and PCR Clean-Up System (Promega), the cloned fragments were sequenced on both strands using BigDye Terminator (Applied Biosystems) according to manufacturer instructions.

### Sequence Analysis of PcP190 Satellite DNA

The obtained nucleotide sequences were edited using the program BioEdit v. 7.0.9.0 (Hall, 1999) and aligned with sequences from GenBank (**Supplementary Table 2**) using the Clustal W algorithm implemented in BioEdit. Similarity analyses were conducted by pairwise comparison using the same program. For identification of PcP190 sequence groups we followed the criteria of Gatto et al. (2016).

### Southern Blot Analysis of PcP190 Satellite DNA

To evaluate the abundance of PcP190 satDNA in male and female *Pseudis bolbodactyla*, *P. cardosoi*, *P. minuta*, *P. paradoxa*, and *L. limellum* (see **Supplementary Table 1** for specimen details), a Southern blot analysis was performed using total genomic DNA samples that were obtained by the standard phenol:chloroform method (Sambrook et al., 1989). The restriction endonucleases used to cut the genomic DNA samples and the PcP190 probes used to detect the restriction fragments were chosen based on the type of PcP190 sequence that was shown to be frequent in each species (**Table 1** summarizes the types of PcP190 sequences found in each species). Enzymes that cleave a single site per monomer of PcP190 sequence were used.

The PcP-2 sequences were digested in their HR by *Mbo*II or *Hind*III, while the PcP-3 sequences had their HR cut by *Hind*III. The PcP-8 sequences were digested in the CR by the endonuclease restriction *Stu*I. Complete genomic DNA digestion was performed with 12 h of endonuclease activity. The restriction fragments were electrophoresed in a 1.2% agarose gel and transferred to a nitrocellulose membrane according to Sambrook et al. (1989). Probes for PcP-2 (Gatto et al., 2016), PcP-3 (PcP190-3-Pmin-M-C8), and PcP-8 (PcP190-8-Llim-M-C7) sequences were labeled by PCR using the PCR Dig Probe Synthesis Kit (Roche) and hybridized overnight at 65°C to the nitrocellulose membrane containing the restriction fragments.

After hybridization, nitrocellulose membranes were washed twice in a solution of 2x SSC/0.1% SDS (5 min each wash) at room temperature and then washed twice in a solution of 0.1x SSC/0.1% SDS (15 min each) at 65°C. Probes were detected using the DIG Nucleic Acid Detection Kit (Roche) following the manufacturer’s instructions.

### Fluorescent *in situ* Hybridization (FISH) of PcP190 Satellite DNA

The PcP-2 sequence PcP190-2-Pbol-F-C7 and PcP-3 sequence PcP190-3-Pmin-M-C5 were labeled using the PCR Dig Probe Synthesis Kit (Roche) to generate PcP-2 and PcP-3 probes, respectively. The resulting amplicons were precipitated in the presence of sonicated salmon sperm DNA (100 ng/μL) and resuspended in hybridization solution (50% formamide, 2x SSC and 10% dextran sulfate). The PcP-2 probe was hybridized to chromosome preparations from *P. bolbodactyla* (four females and four males) and *P. paradoxa* (four females and three males), whereas the PcP-3 probe was hybridized to karyotypes from *P. minuta* (two females and three males) and *P. cardosoi* (two females and two males) (see **Supplementary Table 1** for specimen details). Hybridization assays were performed according to Viegas-Péquignot (1992). Chromosome preparations were incubated at 37°C for 45 min with rhodamine conjugated anti-digoxigenin (600 ng/μL) for probe detection, washed with PBT solution (1x PBS, 0.4% BSA and 0.1% Tween 20) and counterstained with 0.5 μg/μL of 4′,6-Diamidino-2-phenylindole (DAPI). Chromosome preparations were observed in an Olympus Bx-60 (Olympus, Japan) fluorescence microscope. Images were captured with a Q-Color 3 digital system and edited only using brightness and contrast options in Adobe Photoshop CS3 (Adobe Systems).

### Comparative Genomic Hybridization (CGH) in *Pseudis bolbodactyla*

Because the analysis of PcP190 sequences showed sex chromosome heteromorphism in *Pseudis bolbodactyla* (see “Results” section), we employed comparative genomic hybridization (CGH) to analyze the hybridization of female-derived DNA probes to female metaphases of this species in the presence of male-derived competitor DNA. Genomic DNA samples (1 μg each) from females of *P. bolbodactyla*, obtained with a standard phenol:chloroform method (Sambrook et al., 1989), were labeled using the Nick Translation Kit (Roche) by incorporation of FITC-12-dUTP (Roche) or Dig-11-dUTP (Roche). Each labeled sample was precipitated and resuspended in hybridization solution (50% formamide, 2x SSC, and 10% dextran sulfate) together with fragmented male genomic DNA from *P. bolbodactyla* used as competitor DNA. Competitor DNA was obtained by boiling male genomic DNA, diluted in 0.3 M NaCl, in an autoclave for 30 min and 1.4 atm/120°C. Samples of the fragmented DNA were submitted to electrophoresis in 1% agarose gel to check for a fragmentation length distribution between 100 and 1,000 bp, purified by phenol:chloroform standard extraction and resuspended in hybridization solution (50% formamide, 2x SSC and 10% dextran sulfate). Probe/competitor DNA proportions for hybridization assays were 1:10. Hybridization assays were performed as previously mentioned on Section “Fluorescent *in Situ* Hybridization (FISH) of PcP190 Satellite DNA,” using chromosome preparations from two *P. bolbodactyla* females (see **Supplementary Table 1** for specimen details).

## Results

### PcP190 Satellite DNA Diversity in *Pseudis* and *Lysapsus*

**FIGURE 2:**
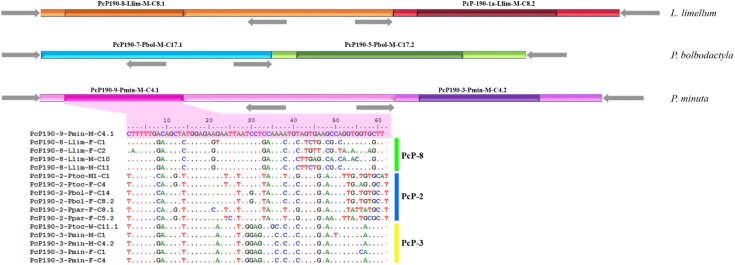
Scheme of fragments isolated from *Lysapsus limellum*, *Pseudis bolbodactyla*, and *Pseudis minuta* composed of different types of juxtaposed PcP190 sequences. Different colors represent distinct types of sequences based on the hypervariable region. The different shades distinguish the hypervariable region (darker shade) from more conserved region (light shade). Gray arrows indicate the P190F and P190R primer sites.

The PCR reactions for PcP190 satDNA isolation generated fragments between 200 and 400 bp, and 69 clones were obtained. Sequences belonging to five of the seven PcP190 groups previously recognized by Gatto et al. (2016) were identified in our sample (**Figure 1** and **Table 1**). Moreover, two new sequence groups were identified based on their HRs: the PcP-8 group for sequences isolated from *Lysapsus limellum* (**Figure 1** and **Table 1**), and the PcP-9 group for one sequence found in *P. minuta* (**Figure 2** and **Table 1**). The average similarity of the HR in each group was above 90%, except for the PcP-2 group (which was 79.83%) (**Table 1**). *Pseudis bolbodactyla* showed the greatest diversity with respect to PcP190 sequences, as sequences belonging to five distinct groups were identified (**Table 1**).

Three of the eight cloned sequences composed of more than one monomer exhibited juxtaposed sequences from different PcP groups. These three multimeric sequences were isolated from *Lysapsus limellum*, *Pseudis bolbodactyla*, and *P. minuta* (**Figure 2**). The heterogeneous multimeric fragment obtained from *P. minuta* (PcP-9 sequence in **Figure 2**) also stands out by its presence of a HR that does not match any of those recognized previously.

Comparisons between the CRs of the PcP190 sequences obtained in this work and sequences from *Pseudis tocantins* (Gatto et al., 2016) and anurans from the genus *Physalaemus*, *Leptodactylus*, and *Crossodactylus* (Vittorazzi et al., 2011, 2014) showed an average similarity of 85.12%.

**FIGURE 3:**
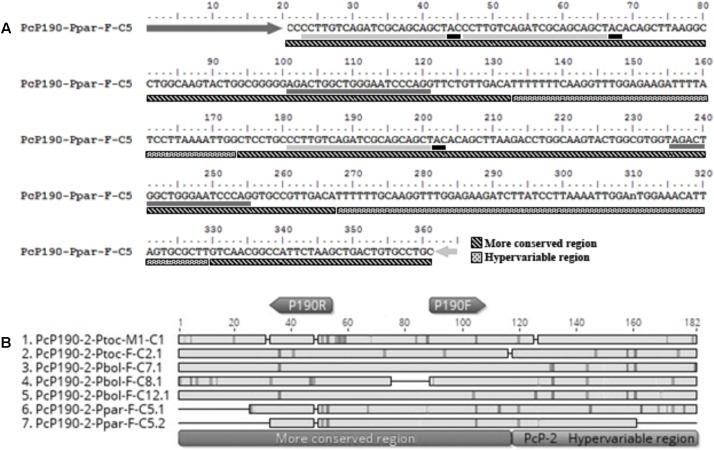
Fragment PcP190-Ppar-F-C5 isolated from *Pseudis paradoxa*. **(A)** Nucleotide sequence. Dark gray and light gray arrows indicate the P190F and P190R primer sites, respectively. Dark gray and light gray underlines indicate regions with similarity to the P190F and P190R primer sites, respectively. Black underlines after P190R primer sites regions indicate the “AC” site normally found after the regions similar to the reverse primer in the PcP190 satellite DNA sequences. **(B)** Alignment of the sequences identified as monomers in the insert PcP190-Ppar-F-C5 with selected PcP-2 sequences of *P. tocantins* and *P. bolbodactyla*. Figure **(B)** was created in Geneious 8.0 (Biomatters).

Another intriguing fragment (PcP190-Ppar-F-C5) was isolated from *Pseudis paradoxa* and included three partial CR intercalated with two HR of the PcP-2 group (**Figure 3**). The first partial CR was 5′-truncated and presented a duplication of the region corresponding to the reverse primer sequence (P190R). These duplicated regions differed from one another by only two sites (**Figure 3**). The second CR was partial due to a deletion of the first 25 bp, and the third CR was 3′-truncated as it ended at the reverse primer incorporated by PCR. One HR in this fragment was 3′-truncated and was only 41 bp, whereas a complete HR with 62 bp was also present (**Figure 3**).

### Southern Blotting Does Not Detect PcP190 satDNA in Male Genome of *Pseudis bolbodactyla*

**FIGURE 4:**
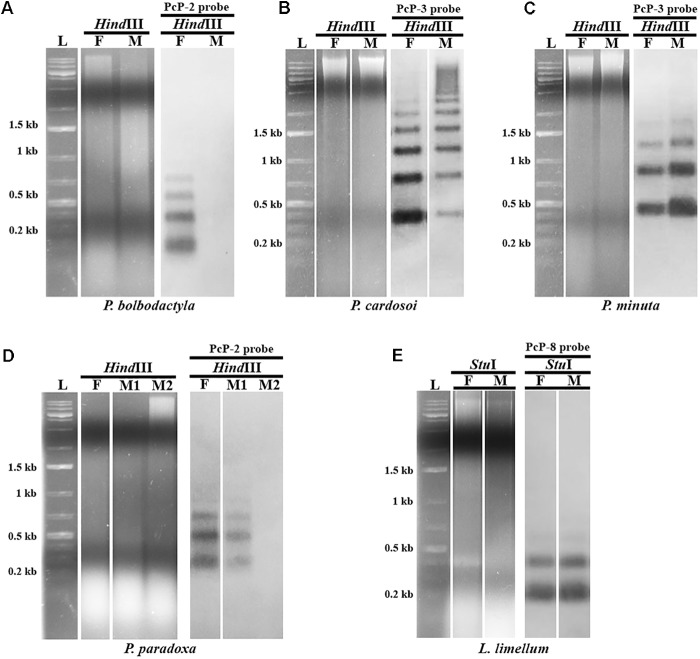
Southern blotting analysis of PcP190 sequences in female (F) and male (M) of *Pseudis bolbodactyla* **(A)**, *P. cardosoi* **(B)**, *P. minuta* **(C)**, *P. paradoxa* **(D)**, and *Lysapsus limellum* **(E)**. The restriction enzyme (*Hind*III or *Stu*I) and the PcP probe for more frequent sequence group in each species (PcP-2, PcP-3, or PcP-8 probe) used in each experiment are indicated. Only one representative blot of each pattern is shown.

Southern blotting analysis of *Pseudis bolbodactyla* female digested genomic DNAs probed with PcP-2 sequences showed a ladder pattern typical of satDNA sequences (**Figure 4A**). However, male genomic DNAs of this species did not show any hybridization signal of the PcP-2 probe in Southern blot, although PcP-1b, PcP-5, and PcP-7 sequences were isolated from the male genome of *P. bolbodactyla* by PCR. In contrast to *P. bolbodactyla*, in *P. paradoxa*, *P. cardosoi*, *P. minuta*, and *L. limellum* the Southern blotting analysis did not show sex-related patterns (**Figures 4B–E**). In the latter three species, both male and female digested genomic DNAs showed the same ladder pattern when hybridized with PcP190 probes (**Figures 4B,C,E**). In *P. paradoxa*, two distinct patterns (i.e., ladder pattern and absence of hybridization signal) were observed both among the three analyzed males and among the four analyzed females (**Figure 4D**).

### PcP190 satDNA Is Mapped to the NOR-Bearing Chromosome Pair in *Pseudis* and Reveals Sex Chromosome Heteromorphism in *Pseudis bolbodactyla*

**FIGURE 5:**
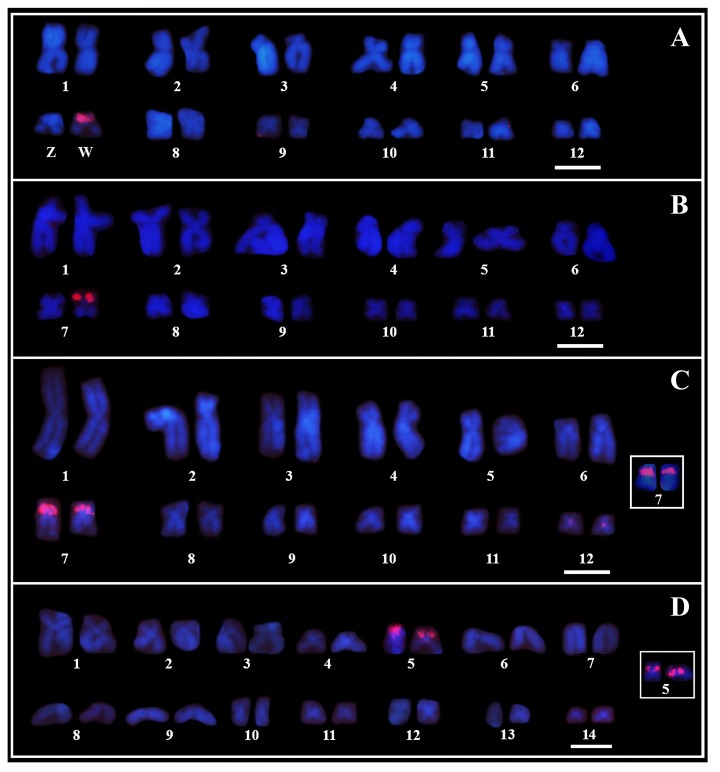
Chromosome mapping of PcP190 satDNA. Fluorescent *in situ* hybridization of PcP-2 **(A, B)** and PcP-3 **(C, D)** probes in female karyotypes of *Pseudis bolbodactyla* **(A)**, *P. paradoxa* **(B)**, *P. minuta* **(C)**, and *P. cardosoi* **(D)**. Insets in **(C, D)** show the pair 7 chromosomes in male *P. minuta* and male *P. cardosoi*, respectively. In *P. paradoxa*, besides the FISH-pattern shown in **(B)**, two females showed no signal of the PcP190 probe in their karyotypes. Bar: 5 μm.

Probes for the PcP190 satDNA showed strong signals of hybridization in the heterochromatic region of the short arm of one of the homologs of pair 7 in the karyotype of four females of *Pseudis bolbodactyla* (**Figure 5A**), while no hybridization signal was observed in the four males of this species (see **Supplementary Table 1** for number of analyzed metaphases). The karyotypes of three males and two females of *P. paradoxa* analyzed showed hybridization signals in one of the homologs of pair 7 (**Figure 5B**). Furthermore, two females of *P. paradoxa* (being one of them not included in the Southern blotting analysis – see **Supplementary Table 1**) did not show any signals of the PcP190 probe in their karyotypes.

In *P. minuta* and *P. cardosoi*, males and females showed the same pattern of hybridization with the PcP190 probes. In both male and female karyotypes of *P. minuta*, the PcP190 satDNA was mapped to the heterochromatic block of the short arm of both pair 7 homologs, and a week hybridization signal of the PcP probes was seen in the centromeric region of chromosome pair 12 (**Figure 5C**). Meanwhile, in *P. cardosoi*, PcP190 satDNA was mapped to the heterochromatic block of the short arm of both pair 5 homologs (**Figure 5D**). For the number of metaphases analyzed per specimen, see **Supplementary Table 1**.

### Sex Chromosome Heteromorphism in *Pseudis bolbodactyla* Revealed by Comparative Genomic Hybridization

**FIGURE 6:**
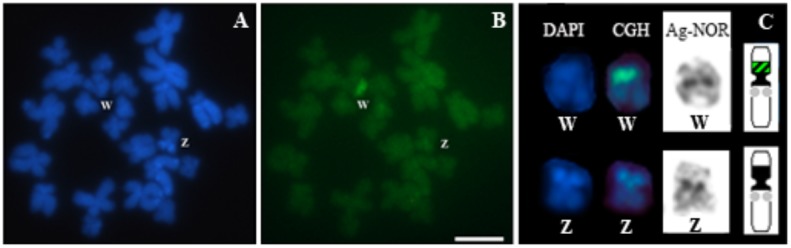
Comparative genomic hybridization (CGH) in a female *Pseudis bolbodactyla*. Chromosome preparations were stained with DAPI **(A)** and submitted to CGH **(B)**. Pair 7 chromosomes from **(A, B)** were sequentially submitted to CGH and the Ag-NOR method, and ideogram of pair 7 is shown **(C)**. In the ideogram in **(C)**, black blocks represent the heterochromatic regions, the NORs sites are indicated by gray circles and the sex-specific site revealed by CGH is shown with dashed black/green. Bar: 5 μm.

Comparative genomic hybridization experiments conducted on chromosome preparations from two females of *Pseudis bolbodactyla* showed a strong hybridization signal of the female-derived probe in exclusively one of the pair 7 homologs (a total of 15 high quality metaphases were analyzed – see **Supplementary Table 1**) (**Figure 6**). The region revealed by CGH corresponds to the pericentromeric heterochromatic block in the short arm of this chromosome, which is also the same region detected by the PcP190 probes (**Figure 5A**).

## Discussion

In this work, we found that PcP190 satDNA is widespread in the genus *Pseudis* and also present in the genus *Lysapsus*. Southern blotting analyses and chromosome mapping showed that PcP190 is sex-related in *Pseudis bolbodactyla.* Chromosome mapping of PcP190 also supports that the sex chromosomes of *P. tocantins* and *P. bolbodactyla* are homeologous to the NOR-bearing chromosomes of the other species of *Pseudis*. Lastly, PcP190 sat DNA mapping allowed us to infer the occurrence of rearrangements involving the NOR-bearing chromosomes of *Pseudis*. In the next sub-sections we discuss these findings in the light of phylogenetic relationships in the genus *Pseudis*.

### PcP190 Satellite DNA

satDNAs usually exhibit species-specific profiles (Tsoumani et al., 2013; Kirov et al., 2017) or are shared by closely related species (Kuhn et al., 2008; Acosta et al., 2010). Few satDNA families are present in phylogenetically distantly related taxa (Robles et al., 2004; Plohl et al., 2010), and the PcP190 satDNA is included in this category, as it occurs in several genera of the anuran superfamily Hyloidea (Vittorazzi et al., 2011, 2014; Gatto et al., 2016).

A high level of homogeneity among the monomers of a given satDNA family is expected because the monomeric units may evolve in concert (reviews in Charlesworth et al., 1994; Ugarković and Plohl, 2002; Plohl et al., 2012). According to the concerted evolution model, the changes in a repetitive unit of a tandem repetitive sequence can be spread to other monomers by a process called molecular drive, which involves unequal crossing-over, gene conversion, transpositions, and insertions of extra chromosome circular DNA (Dover, 1982, 1986; Plohl et al., 2012). However, in *Pseudis tocantins* seven sequence groups of PcP190 satellite sequences were identified, which could be distinguished from each other mainly by their hypervariable region, suggesting that the homogenization mechanisms are not so effective in this case (Gatto et al., 2016). In the present work, different sequence groups of PcP190 were also recognized for *P. minuta*, *Lysapsus limellum*, and particularly *P. bolbodactyla*, including the description of two new sequence groups (PcP-8 in *L. limellum* and PcP-9 in *P. minuta*).

Despite the variation observed in the PcP190 family, we found evidence of rearrangement involving different PcP groups. One line of evidence refers to the juxtaposition of sequences that belong to different PcP-groups, as observed in *Pseudis tocantins* (Gatto et al., 2016), *P. bolbodactyla*, *P. minuta*, and *Lysapsus limellum* (present work). In addition, a cloned fragment of the PcP-2 group, isolated from *P. paradoxa* (PcP190-Ppar-F-C5), showed evidence of rearrangements involving the CR of the PcP190 satDNA, since the CR segment flanked by HR sequences showed a deletion of 25 bp. Moreover, the duplicated regions that correspond to the reverse primer (P190R) site, observed in the 5′ end of this fragment, may also be interpreted as a rearranged segment. That is because we found the dinucleotide “AC” between these two regions that were similar to the reverse primer site, which is the same dinucleotide that follows the reverse primer site in the CR of the PcP-2 group. Due to the presence of this “AC” dinucleotide, it is unlikely that the arrangement in PcP190-Ppar-F-C5 was formed by primer ligation before the cloning procedure, and one might suggest that a rearrangement could be responsible from the deletion of a segment between two reverse primer site regions.

The Southern blotting analysis of PcP190 sequences showed a sex-specific profile in *P. bolbodactyla*, with PcP190 bands detected only in female genomes, as was also reported for *P. tocantins* (Gatto et al., 2016). Because PcP190 sequences (belonging to PcP-1b, PcP-5, and PcP-7) were isolated from the male *P. bolbodactyla* genome, we may conclude that PcP190 sequences are not exclusive, but rather that they are amplified in female *P. bolbodactyla* when compared to male genome of this species, similarly to the situation previously observed in *P. tocantins* (Gatto et al., 2016). The detection of a cluster of PcP190 sequences exclusively in only one chromosome of the female karyotype of *P. bolbodactyla* is in agreement with such an interpretation (see further discussion in the next section). In contrast, in *P. paradoxa*, *P. cardosoi*, *P. minuta*, and *L. limellum*, the Southern blotting showed no sex-specific pattern.

### Sex Chromosomes in *Pseudis*

Busin et al. (2008) proposed, based on the presence of NOR and chromosome morphology, that the sex chromosome pair of *Pseudis tocantins* is homeologous to chromosome pair 5 of *P. cardosoi* and chromosome pair 7 of the other species of *Pseudis*. The mapping of PcP190 satDNA in the heterochromatin of the short arm of chromosome 7 of *P. bolbodactyla*, *P. paradoxa*, and *P. minuta* and chromosome 5 of *P. cardosoi* corroborates this hypothesis of homeology.

In *Pseudis bolbodactyla*, a cluster of PcP190 sequences was detected exclusively in one of the homologs of pair 7 of the female karyotype (all females showed positive hybridization signals) in a site that coincides with that revealed by CGH as the female-specific region. This sex chromosome heteromorphism had not previously been detected by classical cytogenetic techniques (Busin et al., 2008) and allowed for the recognition of the chromosome that bears the PcP190 cluster as the W chromosome of *P. bolbodactyla*. The presence of a PcP190 cluster in the W chromosome and its absence in the Z chromosome of *P. bolbodactyla* (all males analyzed showed no hybridization signals) indicates that the involvement of this satDNA in sex chromosome differentiation is not restricted to *P. tocantins*, although the Wq PcP190 site of *P. tocantins* is much larger than that observed in Wp (W chromosome short arm) of *P. bolbodactyla*.

**FIGURE 7:**
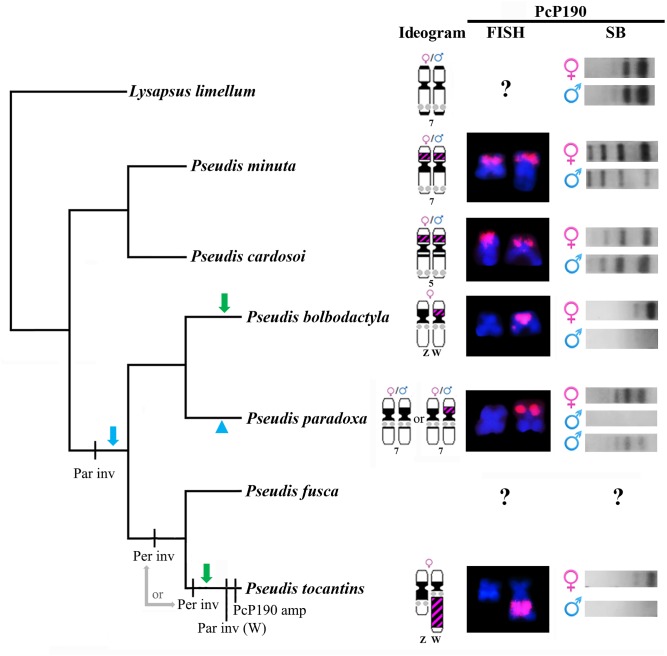
Evolutionary hypotheses relative to differentiation of pair 7 in the genus *Pseudis*, inferred by tracking the cytogenetic data obtained in this work or from Busin et al. (2008) and Gatto et al. (2016) in a species tree. The cladogram shows the phylogenetic relationships of *Pseudis* as inferred by Duellman et al. (2016). FISH: chromosomes hybridized with PcP190 probe. For *P. paradoxa*, only the heterozygous condition for the presence of the PcP190 site is shown in the FISH column. SB: Southern blotting patterns. Par inv: paracentromeric inversion that moved the NOR to a region closer to the centromere. Per inv: pericentromeric inversion that moved the PcP190 site to the long arm; it may have happened in the common ancestor of *P. tocantins* + *P. fusca* or in the linage that gave rise to *P. tocantins*). Par inv (W): paracentromeric inversion in the long arm of chromosome W. PcP190 amp: PcP190 amplification. The green arrows indicate the independent emergence of a sex chromosome heteromorphism related to the PcP190 satDNA (loss of a PcP190 cluster in the Z chromosome) in *P. bolbodactyla* and *P. tocantins*, and an alternative hypothesis is indicated by the blue arrow and arrowhead, which includes the emergence of a sex-related condition regarding the PcP190 satDNA in the common ancestor of *P. bolbodactyla*, *P. paradoxa*, *P. fusca*, and *P. tocantins*, and the loss of this condition in *P. paradoxa*. See text for details.

Meanwhile, in contrast to *Pseudis tocantins* (Gatto et al., 2016) and *P. bolbodactyla*, the mapping of PcP190 satDNA in *P. paradoxa*, *P. minuta*, and *P. cardosoi* karyotypes did not reveal any sex chromosome heteromorphism. Southern blotting analysis confirmed the absence of sex variation related to PcP190 sequences in these three species of *Pseudis* and also in *Lysapsus limellum*. Taken these data together, and considering the phylogenetic relationships in the genus *Pseudis* (Wiens et al., 2010; Duellman et al., 2016), two equally parsimonious hypotheses arise: (i) the sex chromosome heteromorphism related to PcP190 satDNA has arisen after the divergence of the clade composed of *P. minuta* and *P. cardosoi* from the clade that includes *P. tocantins* and *P. bolbodactyla* (which also comprises *P. fusca* and *P. paradoxa*), with the loss of a PcP190 cluster in the Z chromosome of the ancestor of the latter clade and a reversion to the ancestral condition (PcP190 satDNA not related to sex heteromorphism) in *P. paradoxa*; (ii) the sex chromosome heteromorphism related to PcP190 satDNA has arisen independently in *P. bolbodactyla* and *P. tocantins* (**Figure 7**). The analysis of *P. fusca*, which is the sister species of *P. tocantins*, would be helpful to assess this question in future.

In addition, the analysis of cytogenetic data in light of the phylogenetic relationships also suggests that the location of the PcP190 site adjacent to the NOR (as observed in *P. tocantins*) is a derived condition in relation to the positioning of the PcP190 site in a chromosome short arm devoid of NOR (as observed in *P. bolbodactyla*, *P. paradoxa*, and *P. minuta*) (**Figure 7**). Therefore, it is likely that the W chromosome of *P. tocantins* has resulted from a pericentromeric inversion that carried the PcP190 site to the long arm, followed by a paracentromeric inversion that leads to NOR being moved to a region closer the centromere and the PcP190 site to a more distal position in the long arm. In addition to this paracentromeric inversion and after the supposed pericentromeric inversion, an amplification of PcP190 sequences likely expanded the size of the heterochromatic band in Wq. Because in *P. fusca*, the sister species of *P. tocantins*, chromosome 7 does not show an amplified heterochromatic band and its NOR is not as close to the centromere as the NOR in the W chromosome of *P. tocantins* (Busin et al., 2008), we may suppose that the inferred paracentromeric inversion in the long arm and PcP190 amplification have occurred in the evolutionary lineage that gave rise to *P. tocantins*. In contrast, based on the available data, we cannot infer whether the abovementioned putative pericentromeric inversion occurred in the *P. tocantins* lineage or in the common ancestor of *P. tocantins* and *P. fusca* (**Figure 7**). Further mapping of PcP190 sequences in the karyotype of *P. fusca* is thereby necessary to better elucidate this question.

Finally, by tracing the NORs in a phylogenetic tree of *Pseudis*, it is possible to infer the occurrence of a paracentromeric inversion involving the NOR in the common ancestor of *Pseudis bolbodactyla*, *P. fusca*, *P. paradoxa*, and *P. tocantins*, which moved the NOR from a distal site to a more proximal region, since *P. minuta*, *P. cardosoi*, and almost all *Lysapsus* species have the NOR more distally located in pair 7 (pair 5 in *P. cardosoi*) (Busin et al., 2001, 2006, 2008; Suárez et al., 2013) (**Figure 7**).

In conclusion, the study of PcP190 satDNA enabled us to detect an early stage of sex chromosome heteromorphism between morphologically identical chromosomes in *Pseudis bolbodactyla* and to suggest that inversions and amplification of PcP190 sequences were involved in the process that leads to the high level of sex chromosome heteromorphism observed in *P. tocantins*.

Inversions and repetitive DNA amplification, as inferred for *Pseudis*, are events that are expected to occur in early stages of sex chromosome differentiation, as they may play an important role in the suppression of recombination between the proto-sex chromosomes (review in Charlesworth et al., 2005). In addition, repetitive DNA is expected to accumulate rapidly after recombination between sex chromosomes is suppressed (Bergero and Charlesworth, 2009), and repetitive DNA accumulation may be related to genetic degeneration of the W or Y chromosomes (Charlesworth et al., 2005). In anurans, some sex chromosomes have been described that show evidence of an inversion, such as *Tomopterna delalandii* (Schmid, 1980) and *Glandirana rugosa* (Nishioka et al., 1993, 1994), and different levels of heterochromatin accumulation have been found, from low-level heterochromatin accumulation in one of the sex chromosomes (as in *Gastrotheca pseustes* in Schmid et al., 1990) to highly heteromorphic sex chromosomes due to the presence of a great amount of heterochromatin (as in *Pristimantis euphronides* and *P. shrevei* in Schmid et al., 2002). In the case of *P. tocantins*, Wq is highly heterochromatic and further studies regarding the gene content in Z and W may be helpful to evaluate whether any evidence of genetic degeneration is already present.

## Data Availability Statement

The DNA sequences generated for this study are available at the GenBank (www.ncbi.nlm.nih.gov/genbank/) and accession numbers can be found in the **Supplementary Table 1**.

## Author Contributions

KG and LL designed the study and wrote the manuscript. KG and JM performed PcP190 isolation, cloning, sequencing, and chromosome mapping. KS performed CGH analysis and revised the manuscript. All authors approved the final version.

## Conflict of Interest Statement

The authors declare that the research was conducted in the absence of any commercial or financial relationships that could be construed as a potential conflict of interest.
