# Supplementary material for: Sex Chromosome Differentiation in the Frog Genus Pseudis Involves Satellite DNA and Chromosome Rearrangements
Source: Front Genet. 2018 Aug 7;9:301. doi: 10.3389/fgene.2018.00301 (PMC6096759; doi:10.3389/fgene.2018.00301)
Supplement: Supplementary file 1 [file Table_1.DOCX]

**Supplementary table 1** Specimens of *Pseudis* and *Lysapsus* used in this work. The specimens used for isolating PcP190 nucleotide sequences by PCR (PCR), Southern blotting analyses (SB), in situ hybridization (FISH) of PcP190 sequences, and comparative genome hybridization (CGH) are indicated. In the latter two cases, the number of metaphases that showed the results described in the text is presented. In addition, the chromosome locations of the PcP190 sites are indicated by the identification of the chromosome arms that bear those sites (Wp: short arm of the W chromosome; 7p: short arm of chromosome 7; 5p: short arm of chromosome 5). Heterozygous and homozygous conditions are also indicated. ZUEC: Museu de Zoologia “Prof. Adão José Cardoso”, Universidade Estadual de Campinas (UNICAMP), Campinas-SP, Brasil. MNRJ: Museu Nacional, Rio de Janeiro-RJ, Brasil.

| **Species/**  **Specimen voucher** | **Sex** | **Specimen locality** | **PCR (GenBank accession**  **number of cloned sequences)** | **SB** | **FISH (number of metaphases/chromosome location of PcP site)** | **CGH (number of metaphases)** |
| --- | --- | --- | --- | --- | --- | --- |
| *Pseudis bolbodactyla* |  |  |  |  |  |  |
| ZUEC 22080 | female | Formosa/GO |  |  | 13/Wp | 6 |
| MNRJ 34033 | female | Quirinópolis/GO |  |  | 4/Wp |  |
| MNRJ 34036 | female | Quirinópolis/GO |  |  | 24/Wp |  |
| MNRJ 34054 | female | Quirinópolis/GO | MH370388 – MH370396 | ladder pattern | 50/Wp | 9 |
| MNRJ 34034 | male | Quirinópolis/GO | MH370397 – MH370402 | no signal | 47/no signal |  |
| MNRJ 34041 | male | Quirinópolis/GO |  |  | 10/no signal |  |
| MNRJ 34045 | male | Quirinópolis/GO |  |  | 14/no signal |  |
| MNRJ 34052 | male | Quirinópolis/GO |  |  | 65/no signal |  |
| *Pseudis cardosoi* |  |  |  |  |  |  |
| ZUEC 11772 | female | São Francisco de Paula/RS |  | ladder pattern | 7/5p5p |  |
| ZUEC 11774 | female | São Francisco de Paula/RS |  | ladder pattern | 10/5p5p |  |
| ZUEC 11776 | female | São Francisco de Paula/RS |  | ladder pattern |  |  |
| ZUEC 11596 | male | São Francisco de Paula/RS |  | ladder pattern | 2/5p5p |  |
| ZUEC 11597 | male | São Francisco de Paula/RS |  | ladder pattern |  |  |
| ZUEC 11598 | male | São Francisco de Paula/RS |  | ladder pattern |  |  |
| ZUEC 11770 | male | São Francisco de Paula/RS |  |  | 10/5p5p |  |
| *Pseudis minuta* |  |  |  |  |  |  |
| ZUEC 11585 | female | Eldorado do Sul/RS | MH370422 – MH370439; MH370441 | ladder pattern | 6/7p7p |  |
| ZUEC 11587 | female | Eldorado do Sul/RS |  | ladder pattern |  |  |
| ZUEC 22096 | female | Eldorado do Sul/RS |  | ladder pattern | 18/7p7p |  |
| ZUEC 11583 | male | Eldorado do Sul/RS | MH370408 – MH370421; MH370440; MH370442 |  |  |  |
| ZUEC 22082 | male | Eldorado do Sul/RS |  | ladder pattern | 16/7p7p |  |
| ZUEC 22083 | male | Eldorado do Sul/RS |  | ladder pattern |  |  |
| ZUEC 22084 | male | Eldorado do Sul/RS |  | ladder pattern | 4/7p7p |  |
| ZUEC 22093 | male | Eldorado do Sul/RS |  |  | 2/7p7p |  |
| *Pseudis paradoxa* |  |  |  |  |  |  |
| ZUEC 11794 | female | Corumbá/MS |  | ladder pattern | 17/7p |  |
| ZUEC 11795 | female | Corumbá/MS |  | ladder pattern |  |  |
| ZUEC 11798 | female | Corumbá/MS |  | ladder pattern | 6/7p |  |
| ZUEC 12829 | female | Bacabal/MA | MH370403 – MH370407 | no signal | 4/no signal |  |
| ZUEC 12830 | female | Bacabal/MA |  |  | 5/no signal |  |
| ZUEC 11793 | male | Corumbá/MS |  | ladder pattern | 4/7p |  |
| ZUEC 11799 | male | Corumbá/MS |  |  | 4/7p |  |
| ZUEC 11803 | male | Corumbá/MS |  |  | 7/7p |  |
| ZUEC 11804 | male | Corumbá/MS |  | no signal |  |  |
| MNRJ 33859 | male | Corumbá/MS |  | no signal |  |  |
| *Lysapsus limellum* |  |  |  |  |  |  |
| ZUEC 12837 | female | Corumbá/MS |  | ladder pattern |  |  |
| ZUEC 12845 | female | Corumbá/MS |  | ladder pattern |  |  |
| ZUEC 12852 | female | Corumbá/MS |  | ladder pattern |  |  |
| MNRJ 34071 | female | Nossa Senhora do Livramento/MT | MH370443 – MH370452 |  |  |  |
| ZUEC 12844 | male | Corumbá/MS |  | ladder pattern |  |  |
| ZUEC 12848 | male | Corumbá/MS |  | ladder pattern |  |  |
| ZUEC 12850 | male | Corumbá/MS |  | ladder pattern |  |  |
| MNRJ 34072 | male | Nossa Senhora do Livramento/MT | MH370453 – MH370456 |  |  |  |
