# Supplementary material for: Sex Chromosome Differentiation in the Frog Genus Pseudis Involves Satellite DNA and Chromosome Rearrangements
Source: Front Genet. 2018 Aug 7;9:301. doi: 10.3389/fgene.2018.00301 (PMC6096759; doi:10.3389/fgene.2018.00301)
Supplement: Supplementary file 2 [file Table_2.DOCX]

**Supplementary table 2**. Sequences of PcP190 satellite DNA from GenBank used in the comparative analysis in the present work.

| **PcP190 sequences** | **GenBank accession numbers** |
| --- | --- |
| *Physalaemus albifrons* | KM361694.1 - KM361698.1 |
| *Physalaemus albonotatus* | KM361689.1 - KM361693.1 |
| *Physalaemus cuvieri* | JF281109 - JF281125 and KM361673.1 - KM361683.1 |
| *Physalaemus centralis* | KM361684.1 - KM361688.1 |
| *Physalaemus ephippifer* | KM361699.1 and KM361700.1 |
| *Physalaemus marmoratus* | KM361701.1 - KM361706.1 |
| *Leptodactylus latrans* | KM361718.1 - KM361724.1 |
| *Crossocadctylus gaudichaudii* | KM361725.1 and KM361726.1 |
| *Pseudis tocantins* PcP-1a | KX170908, KX170909, KX170931, KX170887, KX170895 and KX170896 |
| *Pseudis tocantins* PcP-1b | KX170911 - KX170920 |
| *Pseudis tocantins* PcP-2 | KX170921 - KX170930 and KX170897 |
| *Pseudis tocantins* PcP-3 | KX170931 - KX170933 |
| *Pseudis tocantins* PcP-4 | KX170887 - KX170889 |
| *Pseudis tocantins* PcP-5 | KX170890, KX170892 and KX170898 |
| *Pseudis tocantins* PcP-6 | KX170891 |
| *Pseudis tocantins* PcP-7a | KX170892 - KX170894 |
| *Pseudis tocantins* PcP-7b | KX170892, KX170895 - KX170898 |
